# Lipid nanoparticle-encapsulated DNA vaccine confers protection against swine and human-origin H1N1 influenza viruses

**DOI:** 10.1128/msphere.00283-24

**Published:** 2024-08-01

**Authors:** The N. Nguyen, Danh C. Lai, Sarah Sillman, Erika Petro-Turnquist, Eric A. Weaver, Hiep L. X. Vu

**Affiliations:** 1 Nebraska Center for Virology, University of Nebraska-Lincoln, Lincoln, Nebraska, USA; 2 School of Veterinary Medicine and Biomedical Sciences and Nebraska Center for Virology, University of Nebraska-Lincoln, Lincoln, Nebraska, USA; 3 School of Biological Sciences, University of Nebraska-Lincoln, Lincoln, Nebraska, USA; 4 Department of Animal Science, University of Nebraska-Lincoln, Lincoln, Nebraska, USA; Johns Hopkins University Bloomberg School of Public Health, Baltimore, Maryland, USA

**Keywords:** lipid nanoparticles, DNA vaccine, influenza A virus, H1N1pdm09

## Abstract

In 2009, a novel swine-origin H1N1 virus emerged, causing a pandemic. The virus, known as H1N1pdm09, quickly displaced the circulating H1 lineage and became the dominant seasonal influenza A virus subtype infecting humans. Human-to-swine spillovers of the H1N1pdm09 have occurred frequently, and each occurrence has led to sustained transmission of the human-origin H1N1pdm09 within swine populations. In the present study, we developed a lipid nanoparticle-based DNA vaccine (LNP-DNA) containing the hemagglutinin gene of a swine-origin H1N1pdm09. In pigs, this LNP-DNA vaccine induced a robust antibody response after a single intramuscular immunization and protected the pigs against challenge infection with the homologous swine-origin H1N1pdm09 virus. In a mouse model, the LNP-DNA vaccine induced antibody and T-cell responses and protected mice against lethal challenge with a mouse-adapted human-origin H1N1pdm09 virus. These findings demonstrate the potential of the LNP-DNA vaccine to protect against both swine- and human-origin H1N1pdm09 viruses.

**IMPORTANCE:**

Swine influenza A virus (IAV) is widespread and causes significant economic losses to the swine industry. Moreover, bidirectional transmission of IAV between swine and humans commonly occurs. Once introduced into the swine population, human-origin IAV often reassorts with endemic swine IAV, resulting in reassortant viruses. Thus, it is imperative to develop a vaccine that is not only effective against IAV strains endemic in swine but also capable of preventing the spillover of human-origin IAV. In this study, we developed a lipid nanoparticle-encapsulated DNA plasmid vaccine (LNP-DNA) that demonstrates efficacy against both swine- and human-origin H1N1 viruses. The LNP-DNA vaccines are non-infectious and non-viable, meeting the criteria to serve as a vaccine platform for rapidly updating vaccines. Collectively, this LNP-DNA vaccine approach holds great potential for alleviating the impact of IAV on the swine industry and preventing the emergence of reassortant IAV strains.

## INTRODUCTION

Influenza A viruses (IAVs) pose a significant public health concern and account for an estimated 3–5 million severe illness cases and approximately 300,000–650,000 deaths worldwide (1). IAVs have a broad host range, infecting various species, including wild waterfowl, domestic poultry, horses, dogs, swine, and humans. The potential for global pandemics arises when influenza viruses originating from animals acquire the ability to infect and transmit among humans (2).

Swine is considered a “mixing vessel” for the emergence of reassortant viruses with pandemic potential due to their susceptibility to both human and avian influenza viruses (3). In 2009, a novel swine-origin H1N1 emerged, marking the first pandemic of the 21st century (4, 5). The genome of the H1N1-pandemic-virus-2009 (H1N1pdm09) is composed of six gene segments (PB2, PB1, PA, HA, NP, and NS) from the North American triple-reassortant swine IAV lineage and two segments (M and NA) from the Eurasian avian-like swine H1N1 lineage (6). H1N1pdm09 rapidly displaced the circulating seasonal H1 lineage and became the dominant IAV subtype infecting humans (7). Human-to-swine transmission of H1N1pdm09 viruses has occurred frequently (8, 9). Once introduced into swine herds, these spillover viruses frequently reassort with endemic swine IAV and continuously adapt in the swine host (10, 11). This adaptation process results in viruses that antigenically drift from the human seasonal H1N1pdm09 (8). Thus, the spillover of IAV from humans to swine significantly influences the evolutionary dynamics of the virus within swine populations.

Given the bidirectional transmission of IAV between humans and swine, effective control measures in swine populations could significantly mitigate the risk of IAV outbreaks in humans. Currently, vaccines containing multiple whole-inactivated virus (WIV) strains are commercially available and widely used to control IAV in swine. However, these WIV vaccines often fail to provide sufficient heterologous protection due to the substantial genetic and antigenic diversity and the emergence of new IAV variants (12, 13). The extensive costs and time required for vaccine licensing hinder prompt updates to match the virus within swine populations.

The Center for Veterinary Biologics, the regulatory authority overseeing veterinary licensing, has issued a memorandum outlining the regulatory framework to facilitate the prompt updating of veterinary vaccine antigens (14). According to these guidelines, if a veterinary vaccine is developed using a non-replicating, non-viable production platform and the initial vaccine product has already been fully licensed, the licensure process for new vaccines containing sequence variants of the same vaccine antigens can be expedited.

Two primary platforms are commonly utilized for producing vaccines against swine IAV. One platform employs protein expression systems, such as the baculovirus expression system, to generate large quantities of hemagglutinin (HA), which is used as the vaccine immunogen. The other platform utilizes replication-defective RNA replicon particles to deliver the HA gene of swine IAV (15, 16). Both the HA protein-based and RNA replicon particle systems have demonstrated efficacy in providing protective immunity against homologous challenge infections in pigs (16–18).

DNA plasmids fulfill regulatory criteria as non-replicating, non-viable vaccine platforms. However, attempts to vaccinate pigs with unencapsulated DNA plasmids encoding the HA gene have had limited success due to the inefficient entry of unencapsulated DNA plasmids into animal cells. Strategies to enhance the efficacy of DNA plasmid vaccines include intradermal administration using needle-free applicators, adsorption onto gold microparticles for epidermal delivery via a gene gun, and *in vivo* electroporation (19–24). Despite these efforts, pigs vaccinated with DNA vaccines containing the HA gene often exhibit low hemagglutinin inhibition (HI) antibody titers and lack protection against IAV challenge, although they may show reduced virus shedding in the nasal cavity (22).

Recently, lipid nanoparticles (LNPs) have emerged as promising nanocarriers for nucleic acid-based vaccines, particularly following the success of the COVID-19 vaccine (reviewed in references 25 and 26). Our recent research has demonstrated that LNP-DNA vaccines containing the H3N2 HA antigen elicited high titers of HI antibodies within 7–14 days post-vaccination in pigs (27). Importantly, these vaccinated pigs were fully protected against challenge infection with the homologous H3N2 virus.

In this study, we developed an LNP-DNA vaccine containing the HA gene of a swine-originating H1N1 pdm09 virus and assessed its immunogenicity in pigs and mice. Animals vaccinated with the LNP-DNA vaccine generated high HI antibody titers against both swine- and human-originating H1N1pdm09 strains. Moreover, pigs vaccinated with this vaccine were protected from challenge infection with the swine-originating H1N1pdm09 strain. Similarly, mice vaccinated with this LNP-DNA vaccine were protected against a lethal challenge with the mouse-adapted human-originating H1N1pdm09 strain. These findings collectively demonstrate that the LNP-DNA vaccine containing the HA gene of a swine-originating H1N1pdm09 virus confers protection against both swine- and human-originating H1N1pdm09 strains.

## MATERIALS AND METHODS

### Cells, viruses, and lipids

HEK-293T (CRL-3216) and Madin-Darby Canine Kidney (MDCK, CCL-34) were obtained from the American Type Culture Collection. The cells were cultured in DMEM supplemented with 10% fetal bovine serum at 37°C with 5% CO_2_. The swine-origin H1pdm09 virus A/swine/Iowa/A01202099/2011 (IA11) was obtained from the National Veterinary Services Laboratories (Ames, IA), and human-origin H1pdm09 virus A/California/07/2009 NR-13663 (CA09) was obtained from Biodefense and Emerging Infections Research Resources (BEI Resources, Manassas, VA). The CA09 virus was adapted to mice using serial lung-to-lung passage as previously described (28). To prepare enough virus stocks for this study, the IA11 virus was passaged once in MDCK cells, while the mouse-adapted CA09 virus was grown in the allantoic cavities of 10-day-old embryonated chicken eggs at 37°C for 48 h. Virus titers were determined by tissue infectious dose 50 (TCID_50_) or hemagglutination units. Additionally, the CA09 virus stock was titrated in BALB/c mice to calculate the 50% mouse lethal dose (MLD_50_).

The peptide array, encompassing the HA protein of the A/New York/18/2009 H1N1pdm09 strain (NY09) of influenza virus, was acquired from BEI Resources (Cat. No. NR-19245). This array comprises 139 peptides, each spanning 15 amino acids with 11 overlaps. It is noteworthy that the HA sequences of NY09 and CA09 exhibit a 99% identity.

Lipids used in this study were DLin-MC3-DMA (MC3) (Nanosoft Polymer, Winston-Salem, NC), 1,2-dioleoyl-3-trimethylammonium-propane (DOTAP) (Cayman Chemical, Ann Arbor, MI), cholesterol (Sigma Aldrich), distearoylphosphatidylcholine (DSPC), and 1,2-dimyristoyl-rac-glycero-3-methoxypolyethylene glycol-2000 (DMG-PEG2000) (Avanti Polar Lipids, Birmingham, AL). The lipids were prepared separately in absolute ethanol.

### DNA plasmid construction

The HA sequence of the IA11 virus (GenBank accession no. JX092275.1) was optimized for expression in swine cells (*Sus scrofa*) using a free online tool provided by GenScript (Piscataway, NJ). The flag-tag sequence (DYKDDDDK) was fused to the carboxyl terminus of the HA gene to facilitate protein detection. The gene fragment was synthesized using a commercial DNA synthesis service (GenScript) and cloned into the pCI plasmid backbone (Promega, Madison, WI). The recombinant plasmid was proliferated in *E. coli* DH5α, and plasmid was purified by using the Giga Prep Kit (Zymo Research, Costa Mesa, CA).

### Preparation of lipid nanoparticles

Lipid nanoparticles encapsulating the DNA plasmid encoding the IA11 HA gene were prepared as previously described (27), with slight modifications. For use in pigs, MC3, DOTAP, DSPC, cholesterol, and DMG-PEG2000 were mixed at a molar ratio of 42:10:8:38:2 to form a mixture with a total lipid concentration of 15 mM*,* and the DNA plasmid was diluted in 25 mM sodium acetate pH 4.0. For use in mice, MC3, DOTAP, DSPC, cholesterol, and DMG-PEG2000 were mixed at a molar ratio of 35:5:10:48:2 to form a mixture with a total lipid concentration of 28 mM, and the DNA plasmid was diluted in 50 mM citrate buffer pH 4.0.

The lipid and DNA solutions were mixed using the Mixer-4 chip and the NanoGenerator Flex-M Nanoparticle Synthesis System (Precigenome, San Jose, CA) with a flow rate ratio of 3 and a total flow rate of 4 mL per minute. The nitrogen-to-phosphate ratio (mol/mol) of the LNP-DNA vaccines used in pigs and mice was 5.5 and 4.5, respectively. The raw products were dialyzed against 100 mM Tris-Cl buffer pH 7.4 using Slide-A-Lyzer G2 dialysis cassettes with a molecular weight cutoff of 10 kDa (Thermo Fisher Scientific, Carlsbad, CA). Following dialysis, LNPs were passed through 0.45 µm PES filters, and the encapsulated plasmid was adjusted to 100 µg/mL.

### LNP characterization

LNP sizes and the polydispersity index (PDI) were determined using the nanoparticle tracking analysis method with the Flow NanoAnalyzer (NanoFCM, Tokyo, Japan). The surface charges of LNPs, expressed as zeta potential (mV), were measured using the Malvern Zetasizer (Nano ZS, Malvern Instrument, Worcestershire, UK). DNA plasmid encapsulation efficiency (EE%) was quantified using a Quant-iT PicoGreen dsDNA Assay Kit (Thermo Fisher Scientific), following the procedure described previously (29).

To assess the stability, the LNP-DNA in Tris-Cl buffer, pH 7.4, was divided into two glass vials; one was stored at room temperature, while the other was stored at 4°C for 8 weeks. Every 2 weeks, a small amount of LNP-DNA solution was removed from the vials for physical characterization. This experiment was repeated twice using two different LNP-DNA preparations.

To assess the transfection efficiency, 500 ng of encapsulated LNP-DNA, either freshly prepared or stored at 4°C or room temperature, was directly added to one well of a 24-well plate containing HEK-293T cells. After 48 h post-transfection, the cells were fixed, and an indirect immunofluorescent assay was performed using the anti-HA monoclonal antibody acquired from BEI resources (Cat. No. NR-28668) (27).

### Pig experiment

Ten weaned pigs, approximately 4 weeks old and seronegative for porcine reproductive and respiratory syndrome virus and influenza A virus, were purchased from Midwest Swine Research. The pigs were randomly divided into two groups of five and housed in two separate rooms in the animal biosafety level 2 research facility at the University of Nebraska-Lincoln. One group received a single intramuscular injection of 500 µg of the LNP-DNA vaccine, while the other group served as a non-vaccination (NV) control. The vaccine was administered via a 20-gauge needle in four locations: two in the neck muscle behind the ears and two in the hamstring muscle. Whole blood samples were collected immediately before and weekly after immunization, and the serum was isolated and stored at −20°C for subsequent evaluation of humoral immune responses.

At 49 days post-vaccination (dpv), all pigs were challenged with 2 × 10^5^ TCID_50_ of the IA11 virus via intratracheal and intranasal routes (27). Nasal swabs were collected daily post-challenge to assess viral shedding. At 5 days post-challenge (dpc), the pigs were humanely euthanized using a fatal dose of sodium pentobarbital. During necropsy, lungs were removed from the chest cavity, and bronchioalveolar lavage fluid (BALF) samples were obtained using 50 mL of cold PBS to measure viral load in the lungs. Gross lung lesions were visually evaluated by a veterinary pathologist who remained blinded to the experimental setup. Samples from the trachea (approximately 1 inch above the carina) and the left apical, middle, and caudal lung lobes were collected and fixed in 10% buffered formalin and processed following routine pathological procedures at the Nebraska Veterinary Diagnostic Center. Lung sections were stained with hematoxylin and eosin (H&E) and evaluated for pathological changes. The parameters for evaluating lung gross and microscopic lesions were described previously (30, 31).

### Mouse experiment

Six-week-old female BALB/c mice were obtained from Charles River Laboratories (Wilmington, MA) and were randomly divided into three treatment groups, each comprising 15 mice. Following a 1-week acclimation period, mice in group 1 and group 2 received an intramuscular injection into the left and right tibialis anterior muscle with 10 µg of encapsulated LNP-DNA (LNP) or 10 µg of un-encapsulated DNA plasmid (UnCap), respectively. Group 3 served as the non-vaccinated control. Whole blood samples were collected from 10 out of 15 mice in each treatment group immediately before and at 28 dpv. Sera were isolated to measure HI antibody titers. At 28 dpv, five mice from each group were humanely euthanized, and their spleens were harvested to evaluate T-cell responses. At 31 dpv, the remaining 10 mice in each group were intranasally challenged with 20 MLD_50_ of the CA09 virus. Five mice were monitored daily for 14 days for weight loss and survival. The mice were euthanized if their weight loss exceeded 25%. The remaining five mice were euthanized at 5 dpc, and their lungs were collected. One-half of the lung was snap frozen and homogenized to measure viral load by RT-q-PCR. The other half was fixed in 10% buffered formalin, paraffin embedded, and sectioned. Lung sections were stained with H&E, and microscopic changes in bronchia/bronchiole and pulmonary parenchyma were evaluated as previously described (32, 33). The grading criteria were as follows: 0  =  no lesions; 1  = mild desquamation of bronchial epithelial cells and inflammatory cell infiltration; 2  = moderate desquamation of bronchial epithelial cells and a moderate level of inflammatory cell infiltration; 3  = severe sloughed bronchial epithelial cells and a severe level of inflammatory cell infiltration.

In addition, the presence of virus-infected cells in both the airway epithelium and pulmonary parenchyma of infected mice was assessed using the *in situ* hybridization (ISH) assay (18). The mean number of virus-infected cells per three 0.25 mm^2^ area was counted and evaluated using a four-point scale: 0 for no signals, 1 for 1–10 positive cells, 2 for 11–30 positive cells, 3 for 31–100 positive cells, and 4 for >100 positive cells.

### Hemagglutination inhibition assay

Mouse and pig sera were heat inactivated and treated with 20% kaolin at a ratio of 1 to 4 for 20 min at room temperature, followed by incubation with an equal volume of 0.5% turkey red blood cells. Treated sera were serially diluted twofold in PBS in a 96-well V-bottom plate, starting with an initial dilution of 1:10 for pig sera and 1:20 for mouse sera. An equal volume of each test virus containing 4 HAU was added to each well, and the plate was then incubated for 30 min. Subsequently, 50 µL of 0.5% turkey red blood cells was added to each well, and the plates were further incubated at room temperature for 1 h. HI titers were defined as the reciprocal dilution of the highest dilution at which hemagglutination was not observed.

### Interferon-γ ELISpot assay

Mouse splenocytes, obtained by gently pressing the mouse spleen against a 70-µm cell strainer, were cryopreserved in RPMI medium containing 45% FBS and 10% DMSO. One day prior to the assay, polyvinylidene fluoride 96-well plates (MilliporeSigma, Temecula, CA) were coated with 50 µL of anti-mouse IFN-γ mAb at a concentration of 5 µg/mL (Mabtech, Cincinnati, OH) overnight at 4°C. Subsequently, the plates were blocked with RPMI supplemented with 10% FBS (cRPMI) at 37°C for 1 h. Revived cryopreserved splenocytes were washed once in cRPMI, resuspended in cRPMI at a density of 5 × 10^6^ cells per milliliter, and 100 µL (containing 5 × 10^5^ cells) was then added to each well of the coated plates. The cells were stimulated with pools of overlapping peptides derived from the HA of the A/NY/18/09 (BEI Resources, Cat. No. NR-19245). Each pool contained approximately 40 peptides at a concentration of 250 ng/peptide/well. The plates were incubated overnight at 37°C in 5% CO_2_. Following incubation, the plates were washed with PBST (PBS containing 0.1% Tween 20, vol/vol) followed by 1 h incubation with 50 µL of biotinylated anti-mouse IFN-γ (clone R4-6A2, Mabtech, Cincinnati, OH) diluted 1:1,000 in PBST. After six washes with PBST, the plates were incubated with 50 µL of streptavidin-conjugated alkaline phosphatase diluted 1:1,000 in PBST for 1 h. After six washes with PBST, spots were developed by adding 100  µL of BCIP/NBT (Plus) alkaline phosphatase substrate (Mabtech, Cincinnati, OH) and incubated for 7 min at room temperature. Subsequently, the plates were washed with distilled water and air dried. Spots were counted using an automated ELISpot plate reader (AID iSpot Reader Spectrum). Data are presented as spot-forming cells per one million splenocytes.

### Quantification of viral RNA copies

To quantify viral loads in nasal swabs and BALF, RNA was extracted from 100 µL of samples using the Quick-RNA Viral Kit (Zymo Research, Costa Mesa, CA), following the manufacturer’s instructions. To quantify viral loads in mouse lungs, the samples were weighed in 1.5 mL tubes and homogenized in 9 (wt/vol) of sterile PBS. Subsequently, 100 µL of the homogenized sample was used for RNA extraction using the Quick-RNA Viral Kit (Zymo Research).

Viral RNA copies were quantified using a commercially available real-time RT-PCR Kit (VetMax-Gold SIV Detection Kit, Life Technologies, Austin, TX). To estimate the viral copies, a standard curve was constructed using 10-fold dilutions of a chemically synthesized RNA fragment with known copy numbers. Data are presented as log_10_ viral RNA copies per 100 µL of nasal swabs or BALF or 1 mg of lung. Samples with a cycle threshold above 38 were considered negative and assigned a value of 0.8 log_10_ RNA copies per 100 µL or 1 mg, corresponding to the lower limit of detection of the assay.

### Statistical analysis

Data are present as the mean and standard error of the mean (SEM). Graphical and statistical analyses were performed using GraphPad Prism 9 (GraphPad Software Inc.; San Diego, CA). HI antibody titers were log_2_ transformed before statistical analysis. In the swine study, continuous data were analyzed using the unpaired *t*-test, whereas ranked data (H&E score) were analyzed using the Mann-Whitney test. In the mouse study, continuous data were analyzed using the ordinary one-way analysis of variance, followed by Tukey’s multiple comparison test. Ranked data were analyzed using the Kruskal-Wallis test, followed by Dunn’s multiple comparisons tests.

## RESULTS

### Physical characteristics and stability of LNP-DNA formulation

**Fig 1:**
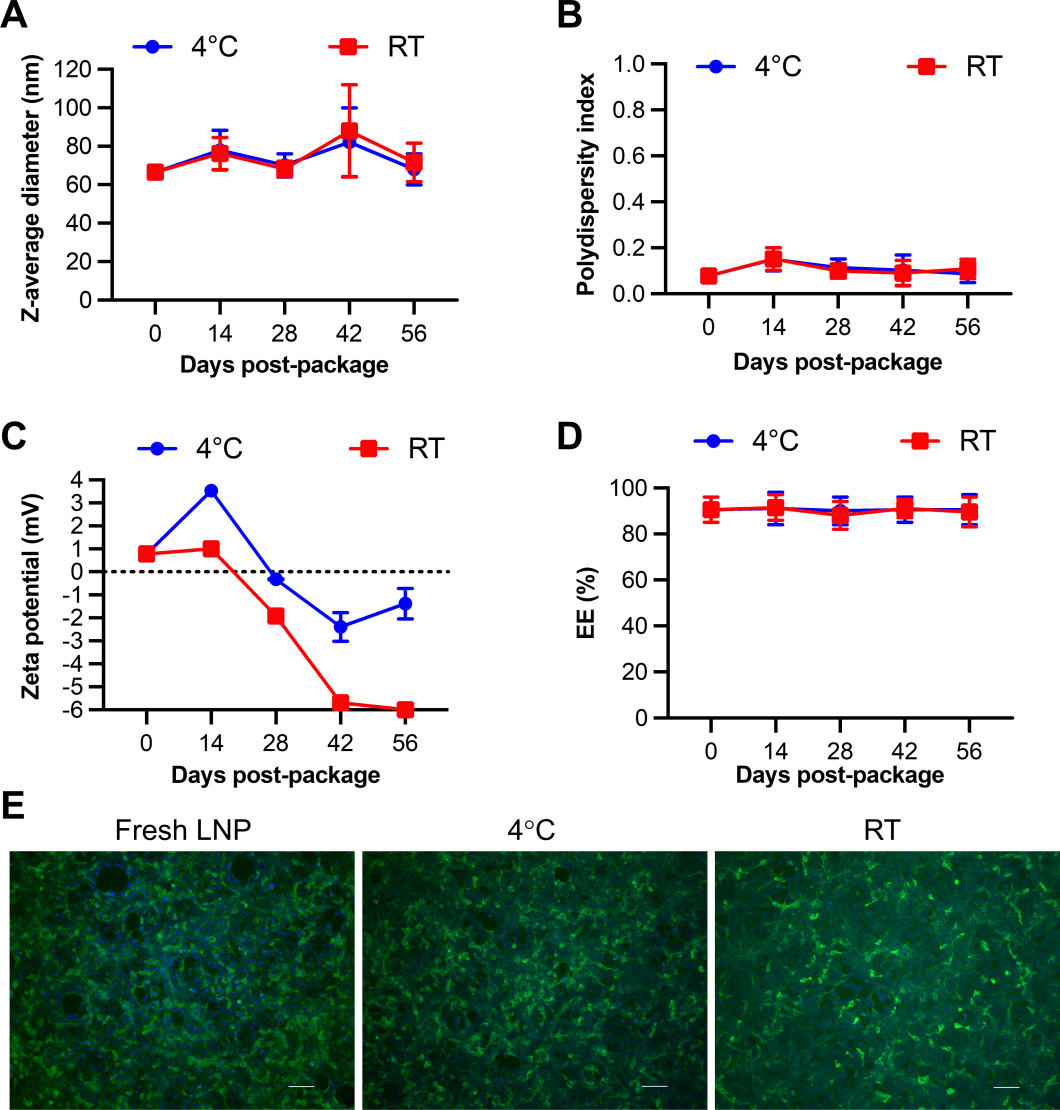
Physicochemical characteristics and the stability of the LNP-DNA vaccine. (**A**) Average diameter, (**B**) polydispersity index, (**C**) zeta potential, and (**D**) encapsulation efficiency of the LNP-DNA freshly prepared or stored at 4°C or room temperature (RT) over 56 days. Data were obtained from two independent LNP-DNA constructs. (**E**) Transfection efficiency in HEK-293T cells of LNP-DNA when freshly prepared or stored at 4°C or room temperature for 56 days. Scale bar = 100 µm. Data presented in this figure were obtained from two independent experiments.

The freshly prepared LNP-DNA vaccine exhibited uniformity in size, with an average diameter of 65 nm and a PDI of 0.1 (Fig. 1A and B). The particles exhibited a neutral surface charge, reflected by a zeta potential of +1 mV (Fig. 1C). Approximately 90% of the DNA plasmid was encapsulated within the LNPs (Fig. 1D). These physical characteristics of the LNP-DNA in the present study closely resemble the findings from our previous investigation involving the H3 antigen (27).

Next, we assessed the stability of LNP-DNA under two storage conditions: room temperature and 4°C. No significant changes were observed in the particle size, polydispersity index, and encapsulation efficiency throughout the 56-day observation period (Fig. 1A, B, and D). However, the zeta potential gradually decreased over time, with the zeta potential of LNP-DNA stored at room temperature exhibiting a greater reduction than stocks stored at 4°C (Fig. 1C).

We assessed the transfection efficacy of the LNP-DNA in HEK-293T cells. The freshly prepared LNP-DNA demonstrated excellent transfection efficiency, as evidenced by the detection of a significant number of cells expressing the H1 protein at 48 hpi (Fig. 1E). The frequencies of H1-expressing cells were remarkably similar in HEK-293T cells transfected with LNP-DNA stored at either room temperature or 4°C for 56 days (Fig. 1E). These findings indicate that the storage conditions did not appear to have any noticeable impact on the transfection efficiency.

### LNP-DNA vaccine elicited a robust antibody response in pigs

**Fig 2:**
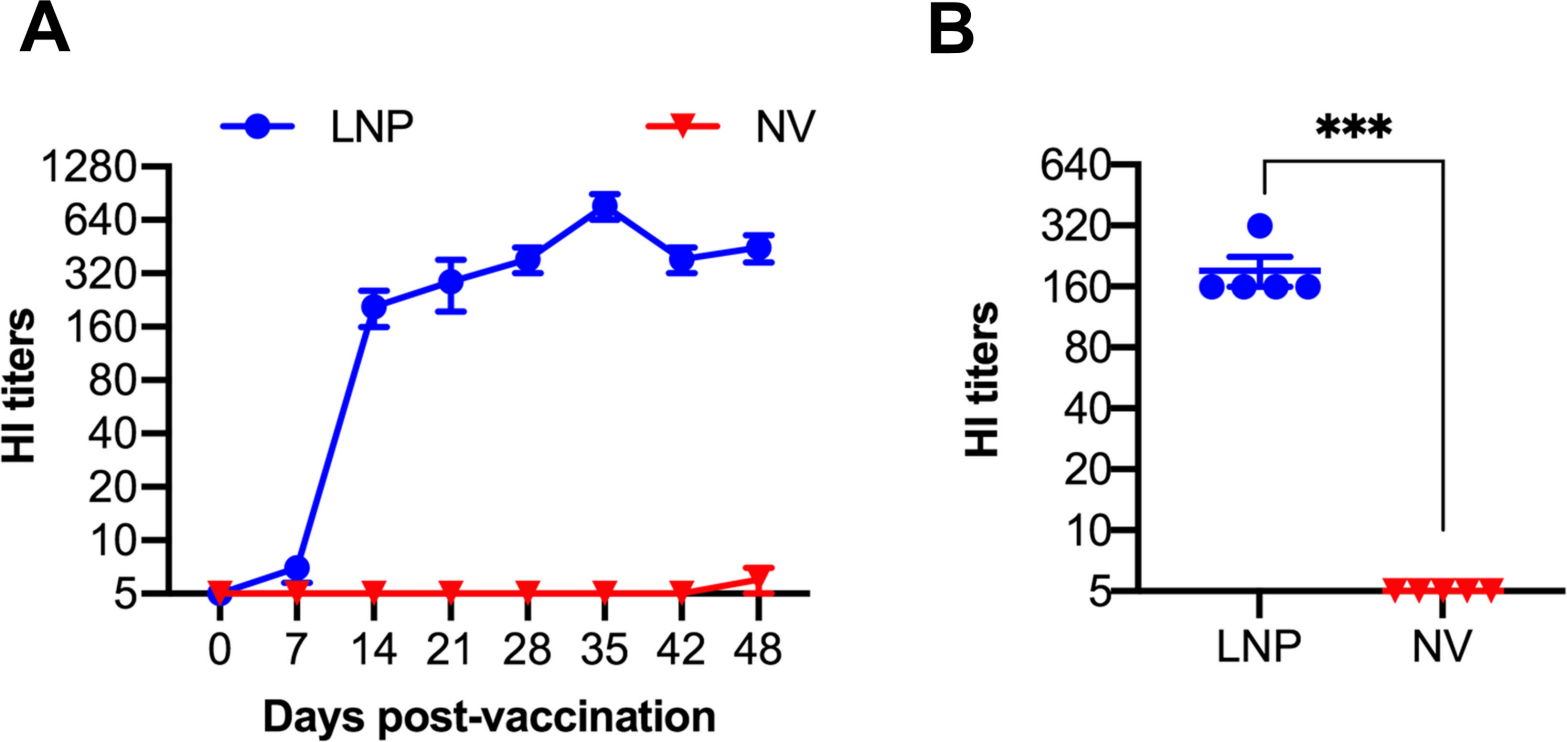
Antibody responses after vaccination. (**A**) Kinetics of HI titers against swine-origin IA11. (**B**) HI titers against human-origin CA09 of serum samples collected at 42 dpv. Samples were initially diluted at 1:10. Samples that showed no HI activity at this initial dilution were assigned a value of 1:5. Data are presented as mean ± SEM. ***P ≤ 0.001.

A vaccination/challenge experiment was conducted in 4-week-old, seronegative pigs to evaluate the immunogenicity and protective efficacy of the LNP-DNA vaccine. The study included two groups of five pigs. One group was injected intramuscularly with 500 µg of the encapsulated LNP-DNA vaccine, while the other group served as non-vaccinated control. In the LNP-DNA group, HI titers against the homologous IA11 virus were first detected at 14 dpv and gradually increased. By 35 dpv, the mean titer reached 1:640, followed by a slight reduction to 1:320 at 48 dpv (Fig. 2A). All pigs in the PBS control group exhibited HI antibody titers below 1:10.

Next, we measured the cross-reactivity against CA09, the human-origin H1N1pdm09. At 42 dpv, sera collected from LNP-DNA-vaccinated pigs exhibited a mean HI titer of 1:160 against the CA09 virus, approximately one log lower than the mean titer against the homologous virus, IA11. As expected, sera collected from the control pigs did not exhibit HI titers against the CA09 virus (Fig. 2B).

### LNP-DNA vaccine conferred protection against the swine-origin H1N1pdm09 virus

**Fig 3:**
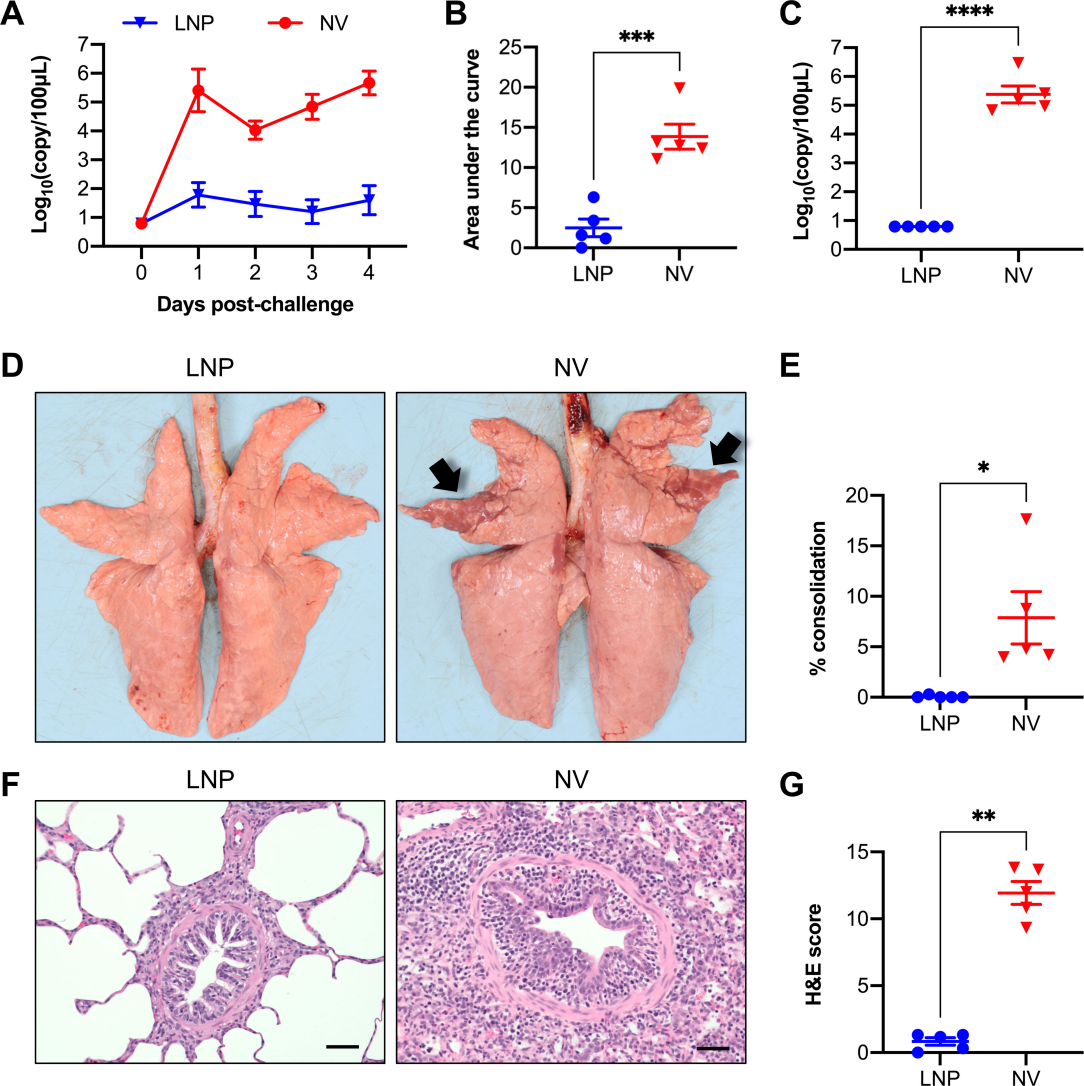
Protection of pigs against challenge with the IA11 virus. (**A**) Viral RNA in nasal swabs detected via RT-q-PCR, presented as log_10_ viral RNA copies per 100 µL sample. (**B**) Area under the curve of viral loads in nasal swabs. (**C**) Viral RNA copies in bronchoalveolar lavage fluid collected on necropsy day. (**D**) Representative lung photos taken during necropsy. Black arrows indicate typical lesions associated with IAV infection. (**E**) Percentage of lung consolidation estimated based on the weighted proportions of each lobe to the total lung volume. (**F**) Representative photo of lung sections stained with H&E. Scale bar = 50 µm. (**G**) Composite microscopic lesion scores. Data are presented as the mean ± SEM *P ≤ 0.05, **P ≤ 0.01, ***P ≤ 0.001, ****P ≤ 0.0001.

At 49 dpv, all pigs were challenged with a swine-originating H1N1pdm09 virus harboring the same HA antigen utilized to prepare the LNP-DNA vaccine. Viral RNA was not detected in swabs collected before the challenge infection (Fig. 3A). Starting from 1 dpc, high viral RNA copy numbers were detected in all pigs of the NV control group. On the other hand, viral RNA was sporadically detected in nasal swabs of the vaccinated pigs, with the highest amount of viral RNA detected being 10^3.1^ copies per 100 µL of the sample. To compare the cumulative difference in viral shedding, the area under the viral shedding curve was calculated. Pigs in the control group exhibited a significantly larger AUC than the LNP-DNA-vaccinated pigs (Fig. 3B).

At 5 dpc, the pigs were euthanized, and BALF was collected to evaluate viral loads within the lungs. High viral RNA copies, up to 10^6^ copies per 100 mL, were detected in the BALF of pigs in the control group, while viral RNA was not detected in the LNP-DNA group (Fig. 3C).

The lungs of pigs in the NV group presented with purple-red consolidation typical of IAV infection, with the percentage of total lung surface consolidation ranging from 4.0% to 17.6% (Fig. 3D and E). In contrast, lung consolidation was not observed in pigs of the LNP-DNA group (Fig. 3D and E). Regarding microscopic lesions, lung sections from pigs in the NV group displayed intense peribronchiolar and perivascular lymphocytic cuffing, necrotic and thinned bronchiolar/bronchial epithelial lining, and suppurative bronchiolitis. Conversely, lung sections of pigs from the LNP-DNA group had minor histopathological changes (Fig. 3F and G). Collectively, these results demonstrate that pigs vaccinated with the LNP-DNA vaccine were fully protected against challenge infection with the homologous IA11 strain.

### LNP-DNA vaccine elicited robust immune responses in mice

**Fig 4:**
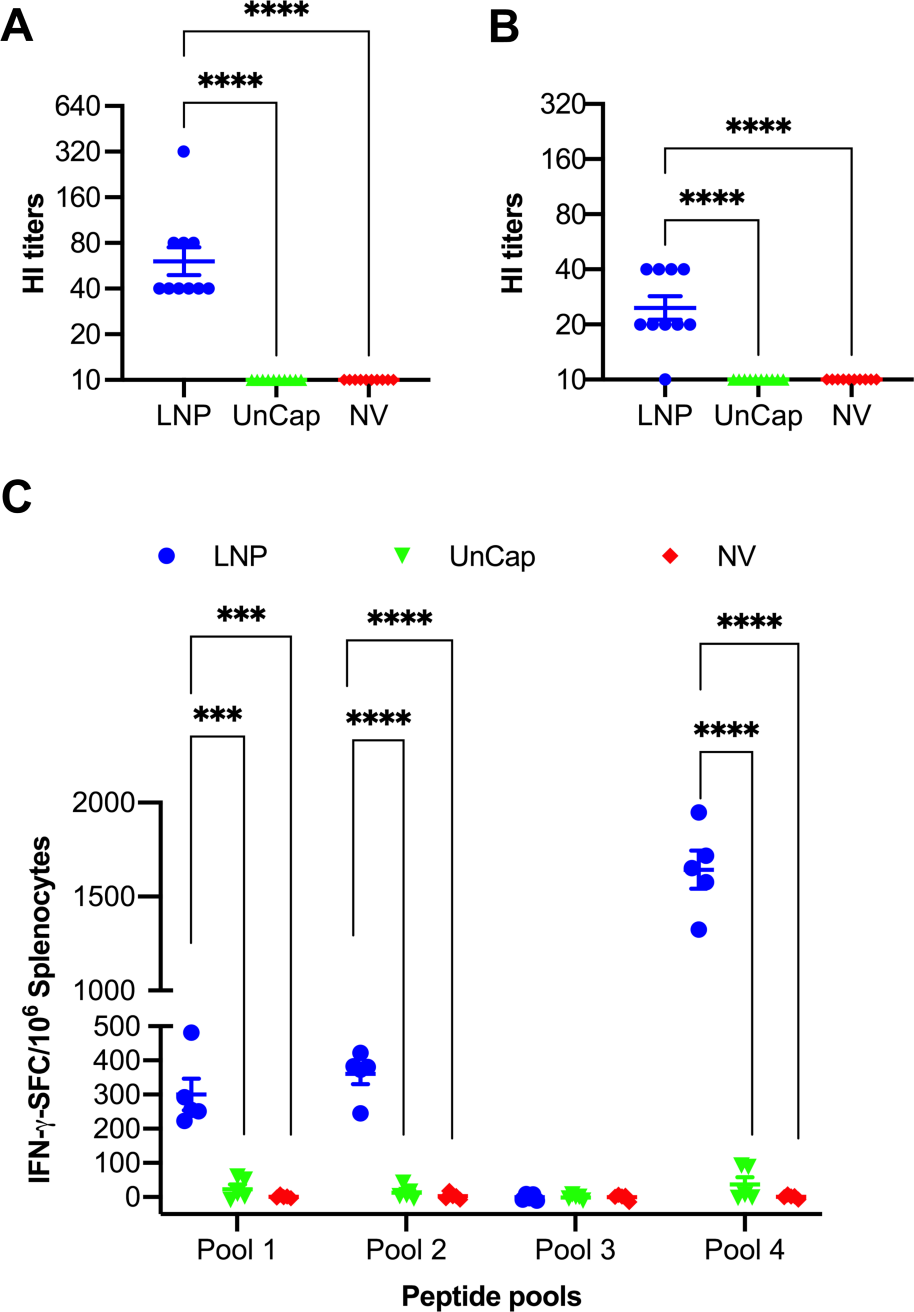
LNP-DNA vaccine elicited robust immune responses in mice. Sera were collected at 28 dpv, and HI titers were measured against IA11 (**A**) and CA09 (**B**) viruses. Samples were initially diluted at 1:20. Samples that showed no HI activity at this initial dilution were assigned a value of 1:10 for statistical purposes. (**C**) The IFN-γ-secreting cells measured against four overlapping peptide pools. Data are presented as mean ± SEM. ***P ≤ 0.001, ****P ≤ 0.0001.

To evaluate the immunogenicity of the LNP-DNA vaccine against a human-origin H1N1pdm09 (CA09) virus, a vaccination and challenge experiment was conducted in mice. At 28 dpv, sera from mice immunized with the LNP-DNA vaccine exhibited antibodies against the IA11 virus, with a mean HI titer of 1:80 (Fig. 4A). Similarly, sera of the LNP-DNA-vaccinated mice displayed HI titers against the CA09 virus, although the mean HI titer was slightly lower than that measured against the IA11 virus (Fig. 4B). Conversely, mice administered the unencapsulated DNA plasmid did not demonstrate detectable HI antibody titers against either IA11 or CA09 viruses.

Next, we assessed T-cell response using the IFN-γ ELISpot assay. Splenocytes were stimulated *ex vivo* with four pools of overlapping peptides derived from the NY09 HA sequence, which has 99% identity to the CA09 HA sequence. Notably, splenocytes collected from mice receiving the LNP-DNA vaccine exhibited high frequencies of IFN-γ spots when stimulated with peptides in pools 1, 2, and 4. In contrast, splenocytes from mice vaccinated with the unencapsulated DNA plasmid displayed minimal frequencies of IFN-γ spots when stimulated with peptides in these pools. Splenocytes from both the LNP-DNA and unencapsulated DNA groups showed no response to the peptides in pool 3 (Fig. 4C).

### LNP-DNA vaccine protected mice against mouse-adapted human-originating H1N1pdm09

**Fig 5:**
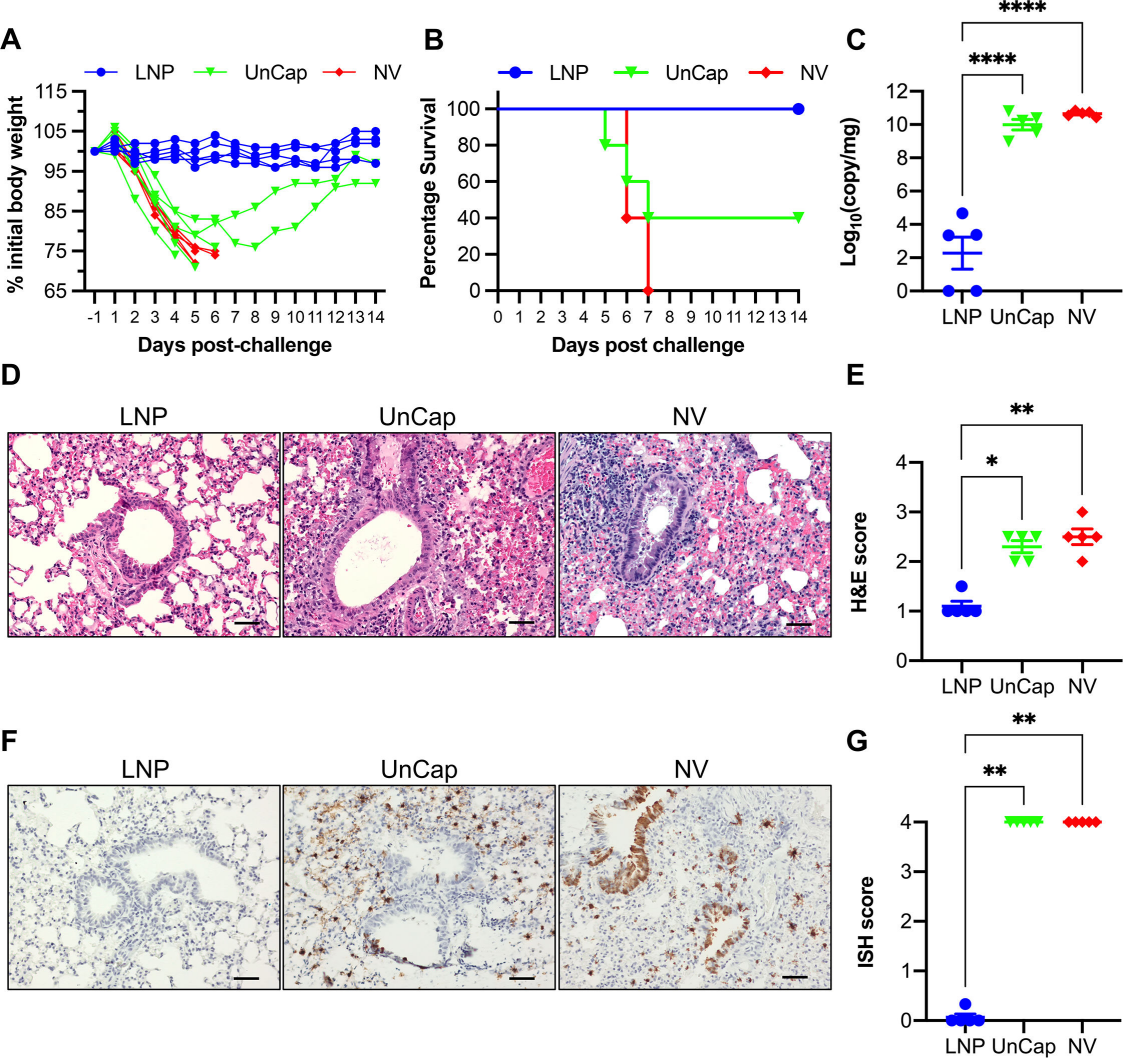
Protection of mice against challenge infection with the CA09 virus. (**A**) Weight loss and (**B**) survival curves of the mice over a 14-day post-challenge observation. (**C**) Viral RNA in the left lung quantified by RT-PCR and presented as log_10_ viral RNA copies per 1 mg of sample. (**D**) Representative photos of lung sections stained with H&E and (**E**) the composite microscopic lesion scores. (**F**) Representative photos of the lung sections stained with ISH to detect viral-infected cells and (**G**) ISH scores. Scale bar = 50 µm. Data are presented as mean ± SEM. *P ≤ 0.05, **P ≤ 0.01, ****P ≤ 0.0001.

To evaluate the protective efficacy of the LNP-DNA vaccination, mice were intranasally challenged with 20 times the lethal dose (20MLD_50_) of the mouse-adapted H1N1pdm09 CA09. Following challenge infection, all mice in the non-vaccinated group experienced severe weight loss and succumbed by 7 dpc (Fig. 5A and B). Mice immunized with unencapsulated DNA plasmid also experienced severe weight loss; however, two out of five mice recovered and survived until the end of the study. Conversely, none of the mice vaccinated with LNP-DNA exhibited weight loss, and they all survived until the end of the 14-day observation period (Fig. 5A and B).

Five mice from each group were euthanized at 5 dpc to assess viral loads in the lungs and lung pathology. High numbers of viral RNA copies, ranging between 10^9^ and 10^11^ copies per milligram of tissue, were detected in the lungs of both non-vaccinated mice and those vaccinated with the unencapsulated DNA plasmid (Fig. 5C). Conversely, viral RNA was not detected in two out of five mice in receiving the LNP-DNA vaccine, while the remaining three mice in this group exhibited minimal numbers of viral RNA copies, ranging from 10^3^ to 10^5^ copies per milligram of tissue (Fig. 5C).

In terms of lung histopathology, the LNP-DNA group exhibited significantly milder lesions than the other groups (Fig. 5D and E). Lung sections from mice in the non-vaccinated and unencapsulated DNA groups exhibited intense infiltration of inflammatory cells in the pulmonary parenchyma, severe peri-bronchiolar inflammation, sloughing of the epithelium, and airway infiltration by mononuclear cells and necrotic debris (Fig. 5D). In contrast, lung sections from mice in the LNP-DNA group displayed minimal microscopic changes.

*In situ* hybridization was utilized to detect viral-infected cells within the lung sections. Numerous ISH-positive cells were detected in alveolar macrophages and bronchiolar epithelium of mice in the control and unencapsulated DNA groups. However, ISH-positive cells were nearly absent in the lungs of mice in the LNP-DNA group (Fig. 5F). Consequently, lung sections of mice in the LNP-DNA group displayed significantly lower ISH scores than those in the other groups (Fig. 5G). Collectively, these data indicate that immunization with the LNP-DNA vaccine can elicit improved protection against heterologous CA09 challenge in mice compared to immunization with unencapsulated DNA alone.

## DISCUSSION

Lipid nanoparticles have proven to be effective carriers for mRNA-based vaccines, as evidenced by the success of COVID-19 vaccines (reviewed in reference 26). In addition to mRNA, LNPs have been utilized to deliver DNA plasmids for gene therapy and immunization. LNP-DNA vaccines encoding antigens from various pathogens, including viruses and bacteria, have demonstrated efficacy across multiple species, including mice, rabbits, hamsters, transchromosomic bovines, and non-human primates (34–38). Our previous research has shown that LNPs effectively delivered DNA plasmid encoding the HA gene of an H3N2 influenza virus strain in pigs (27). This study further demonstrates that the same LNP formulation is effective in inducing immunity against H1N1pdm09 in both pigs and mice. Therefore, accumulating evidence suggests that LNP-DNA represents a versatile platform for inducing protective immunity across various animal species.

In our previous study, pigs that received a single immunization dose with LNP-DNA encoding the HA gene of the H3N2 virus displayed a robust immune response, with HI antibody titers ranging between 1:640 and 1:1,280 at 35 dpv (27). In this study, pigs vaccinated with a single dose of the LNP-DNA vaccine encoding the HA gene of IA11 also exhibited robust HI antibody titers, peaking at 1:640 at 35 dpv and declining to 1:320 at 48 dpv. The results support the notion that LNP-DNA vaccine effectiveness is not immunogen specific (36). In our prior study, pigs inoculated with the LNP-DNA vaccine exhibited sterilizing immunity upon challenge infection with the homologous H3N2 virus. In the current study, viral RNA was sporadically detected in nasal swabs of vaccinated pigs after challenge infection, with the highest amount of viral RNA detected being 10^3.1^ copies per 100 µL of the sample. This discrepancy could be attributed to the delayed challenge infection of the vaccinated pigs in this study compared to the previous study (e.g., 49 vs 35 dpv) and the use of different influenza virus strains (H1N1pdm09 vs H3N2). Based on our experience, samples with such low amounts of viral RNA loads were not infectious when virus isolation assays were attempted on MDCK cells (17). Therefore, we suspect that the pigs would not transmit the virus to other pigs.

For IAV, the HI antibody titer serves as a reliable correlate of vaccine-induced protection. Sera obtained from pigs vaccinated with the IA11-based LNP-DNA vaccine displayed cross-HI titers against the CA09 virus. These findings suggest that the immune responses against the IA11 HA antigen (swine origin) may confer protection against CA09 virus (human origin). To substantiate this hypothesis, we employed a mouse model to assess the protective efficacy of the IA11-based LNP-DNA vaccine against CA09, as mice are more cost-effective than pigs. All vaccinated mice survived and did not exhibit any signs of body weight loss. Additionally, only minimal viral RNA was detected in lung homogenates collected at 5 dpc. The data from the mouse study revealed that the IA11-based LNP-DNA vaccine conferred protection against the CA09 challenge. The CA09 virus was isolated from one of the first human cases in 2009 (39), while IA11 was discovered in swine 2 years later, in 2011. Our findings that the LNP-DNA vaccine based on IA11 provided protection against both IA11 and CA09 are significant. They suggest that the LNP-DNA vaccine formulation could potentially reduce the transmission of human-origin H1N1pdm09 to swine, thereby limiting the opportunities for the virus to evolve and adapt in the swine host.

Immunization of mice with unencapsulated DNA plasmid encoding the HA gene of IAV has been shown to induce protective immunity but often requires multiple doses (40). In this study, the unencapsulated DNA plasmid was administered as a single dose, and it did not induce detectable HI antibodies or IFN-γ-secreting cells. Conversely, a single immunization with the LNP-DNA vaccine, containing the same amount of DNA plasmid, resulted in robust HI antibody titers and IFN-γ-secreting cells. Our findings provide direct evidence that encapsulating DNA plasmids with LNPs enhances their immunogenicity. This enhancement is likely attributed to augmented plasmid uptake, leading to increased production of encoded gene products, as observed in previous studies (36).

For veterinary applications, vaccines that do not require stringent storage conditions, such as ultralow temperatures, are more practical. In our studies, freshly prepared LNP-DNA vaccines were used for animal vaccination. However, we obtained evidence that the LNP-DNA vaccine can be stored at room temperature and at 4°C for 8 weeks while maintaining physical parameters and transfection efficiency comparable to freshly prepared LNP-DNA preparations. While these *in vitro* assessments do not guarantee that LNP-DNA vaccines will retain the ability to induce immunity when administered to animals, they suggest its potential effectiveness. Further research is needed to assess the immunogenicity of LNP-DNA vaccine storage under various conditions.

DNA plasmids exhibit high stability and can be produced in large quantities at low cost. The ability of LNP-DNA to elicit protective immunity with a single dose of immunization would significantly reduce the labor needed to administer the vaccine. Moreover, the vaccinated animals would develop immunity sooner, thereby narrowing the window of susceptibility to infection. Collectively, LNP-DNA holds great potential as a versatile platform for the development of vaccines against influenza viruses in swine.

## Data Availability

All data necessary to support the main findings are included in this paper.

## References

[1] WHO. 2023. Influenza (Seasonal). Available from: https://wwwwhoint/news-room/fact-sheets/detail/influenza-(seasonal). Retrieved 25 Sep 2023.

[2] Pike BL, Saylors KE, Fair JN, Lebreton M, Tamoufe U, Djoko CF, Rimoin AW, Wolfe ND. 2010. The origin and prevention of pandemics. Clin Infect Dis 50:1636–1640. doi:10.1086/652860 20450416 PMC2874076

[3] Ma W, Kahn RE, Richt JA. 2008. The pig as a mixing vessel for influenza viruses: human and veterinary implications. J Mol Genet Med 3:158–166. doi:10.4172/1747-0862.1000028 19565018 PMC2702078

[4] Novel Swine-Origin Influenza A, Dawood FS, Jain S, Finelli L, Shaw MW, Lindstrom S, Garten RJ, Gubareva LV, Xu X, Bridges CB, Uyeki TM. 2009. Emergence of a novel swine-origin influenza A (H1N1) virus in humans. N Engl J Med 360:2605–2615. doi:10.1056/NEJMoa0903810 19423869

[5] Smith GJD, Vijaykrishna D, Bahl J, Lycett SJ, Worobey M, Pybus OG, Ma SK, Cheung CL, Raghwani J, Bhatt S, Peiris JSM, Guan Y, Rambaut A. 2009. Origins and evolutionary genomics of the 2009 swine-origin H1N1 influenza A epidemic. Nature 459:1122–1125. doi:10.1038/nature08182 19516283

[6] Garten RJ, Davis CT, Russell CA, Shu B, Lindstrom S, Balish A, Sessions WM, Xu X, Skepner E, Deyde V, et al.. 2009. Antigenic and genetic characteristics of swine-origin 2009 A(H1N1) influenza viruses circulating in humans. Science 325:197–201. doi:10.1126/science.1176225 19465683 PMC3250984

[7] Quéromès G, Frobert E, Burtseva E, Drăgănescu A, Koul PA, Komissarov A, Laguna-Torres VA, Leblanc J, López-Labrador F-X, Medić S, Mironenko A, Otieno NA, Ruiz-Palacios GM, Md T, Ngs T-L, Josset L, Lina B, Gihsn Collaborators. 2022. Clinical and phylogenetic influenza dynamics for the 2019-20 season in the global influenza hospital surveillance network (GIHSN) - Pilot study. J Clin Virol 152:105184. doi:10.1016/j.jcv.2022.105184 35594785

[8] Markin A, Ciacci Zanella G, Arendsee ZW, Zhang J, Krueger KM, Gauger PC, Vincent Baker AL, Anderson TK. 2023. Reverse-zoonoses of 2009 H1N1 pandemic influenza A viruses and evolution in United States swine results in viruses with zoonotic potential. PLoS Pathog 19:e1011476. doi:10.1371/journal.ppat.1011476 37498825 PMC10374098

[9] Nelson MI, Stratton J, Killian ML, Janas-Martindale A, Vincent AL. 2015. Continual reintroduction of human pandemic H1N1 influenza A viruses into swine in the United States, 2009 to 2014. J Virol 89:6218–6226. doi:10.1128/JVI.00459-15 25833052 PMC4474294

[10] Ryt-Hansen P, Krog JS, Breum SØ, Hjulsager CK, Pedersen AG, Trebbien R, Larsen LE. 2021. Co-circulation of multiple influenza A reassortants in swine harboring genes from seasonal human and swine influenza viruses. Elife 10:e60940. doi:10.7554/eLife.60940 34313225 PMC8397370

[11] Rajão DS, Walia RR, Campbell B, Gauger PC, Janas-Martindale A, Killian ML, Vincent AL. 2017. Reassortment between swine H3N2 and 2009 pandemic H1N1 in the United States resulted in influenza A viruses with diverse genetic constellations with variable virulence in pigs. J Virol 91:e01763-16. doi:10.1128/JVI.01763-16 27928015 PMC5286888

[12] Van Reeth K, Van Gucht S, Pensaert M. 2003. Investigations of the efficacy of European H1N1- and H3N2-based swine influenza vaccines against the novel H1N2 subtype. Vet Rec 153:9–13. doi:10.1136/vr.153.1.9 12877210

[13] Vincent AL, Ciacci-Zanella JR, Lorusso A, Gauger PC, Zanella EL, Kehrli ME, Janke BH, Lager KM. 2010. Efficacy of inactivated swine influenza virus vaccines against the 2009 A/H1N1 influenza virus in pigs. Vaccine 28:2782–2787. doi:10.1016/j.vaccine.2010.01.049 20132919

[14] CVB. 2018. Veterinary services memorandum no. 800.213. https://wwwaphisusdagov/animal_health/vet_biologics/publications/memo_800_213pdf.

[15] Erdman MM, Kamrud KI, Harris DL, Smith J. 2010. Alphavirus replicon particle vaccines developed for use in humans induce high levels of antibodies to influenza virus hemagglutinin in swine: proof of concept. Vaccine 28:594–596. doi:10.1016/j.vaccine.2009.10.015 19853679

[16] Vander Veen RL, Loynachan AT, Mogler MA, Russell BJ, Harris DLH, Kamrud KI. 2012. Safety, immunogenicity, and efficacy of an alphavirus replicon-based swine influenza virus hemagglutinin vaccine. Vaccine 30:1944–1950. doi:10.1016/j.vaccine.2012.01.030 22269873

[17] Kumari S, Chaudhari J, Huang Q, Gauger P, De Almeida MN, Liang Y, Ly H, Vu HLX. 2022. Immunogenicity and protective efficacy of a recombinant pichinde viral-vectored vaccine expressing influenza virus hemagglutinin antigen in pigs. Vaccines (Basel) 10:1400. doi:10.3390/vaccines10091400 36146478 PMC9505097

[18] Sun H, Sur JH, Sillman S, Steffen D, Vu HLX. 2019. Design and characterization of a consensus hemagglutinin vaccine immunogen against H3 influenza A viruses of swine. Vet Microbiol 239:108451. doi:10.1016/j.vetmic.2019.108451 31767095

[19] Gorres JP, Lager KM, Kong WP, Royals M, Todd JP, Vincent AL, Wei CJ, Loving CL, Zanella EL, Janke B, Kehrli ME, Nabel GJ, Rao SS. 2011. DNA vaccination elicits protective immune responses against pandemic and classic swine influenza viruses in pigs. Clin Vaccine Immunol 18:1987–1995. doi:10.1128/CVI.05171-11 21918118 PMC3209026

[20] Borggren M, Nielsen J, Karlsson I, Dalgaard TS, Trebbien R, Williams JA, Fomsgaard A. 2016. A polyvalent influenza DNA vaccine applied by needle-free intradermal delivery induces cross-reactive humoral and cellular immune responses in pigs. Vaccine 34:3634–3640. doi:10.1016/j.vaccine.2016.05.030 27211039 PMC4940207

[21] Bragstad K, Vinner L, Hansen MS, Nielsen J, Fomsgaard A. 2013. A polyvalent influenza A DNA vaccine induces heterologous immunity and protects pigs against pandemic A(H1N1)pdm09 virus infection. Vaccine 31:2281–2288. doi:10.1016/j.vaccine.2013.02.061 23499598

[22] Karlsson I, Borggren M, Rosenstierne MW, Trebbien R, Williams JA, Vidal E, Vergara-Alert J, Foz DS, Darji A, Sisteré-Oró M, Segalés J, Nielsen J, Fomsgaard A. 2018. Protective effect of a polyvalent influenza DNA vaccine in pigs. Vet Immunol Immunopathol 195:25–32. doi:10.1016/j.vetimm.2017.11.007 29249314 PMC5764121

[23] Macklin MD, McCabe D, McGregor MW, Neumann V, Meyer T, Callan R, Hinshaw VS, Swain WF. 1998. Immunization of pigs with a particle-mediated DNA vaccine to influenza A virus protects against challenge with homologous virus. J Virol 72:1491–1496. doi:10.1128/JVI.72.2.1491-1496.1998 9445052 PMC124630

[24] Larsen DL, Olsen CW. 2002. Effects of DNA dose, route of vaccination, and coadministration of porcine interleukin-6 DNA on results of DNA vaccination against influenza virus infection in pigs. Am J Vet Res 63:653–659. doi:10.2460/ajvr.2002.63.653 12013464

[25] Parhiz H, Atochina-Vasserman EN, Weissman D. 2024. mRNA-based therapeutics: looking beyond COVID-19 vaccines. Lancet 403:1192–1204. doi:10.1016/S0140-6736(23)02444-3 38461842

[26] Hald Albertsen C, Kulkarni JA, Witzigmann D, Lind M, Petersson K, Simonsen JB. 2022. The role of lipid components in lipid nanoparticles for vaccines and gene therapy. Adv Drug Deliv Rev 188:114416. doi:10.1016/j.addr.2022.114416 35787388 PMC9250827

[27] Nguyen TN, Kumari S, Sillman S, Chaudhari J, Lai DC, Vu HLX. 2023. A single-dose intramuscular immunization of pigs with lipid nanoparticle DNA vaccines based on the hemagglutinin antigen confers complete protection against challenge infection with the homologous influenza virus strain. Vaccines (Basel) 11:1596. doi:10.3390/vaccines11101596 37896997 PMC10611089

[28] Xu L, Bao L, Li F, Lv Q, Ma Y, Zhou J, Xu Y, Deng W, Zhan L, Zhu H, Ma C, Shu Y, Qin C. 2011. Adaption of seasonal H1N1 influenza virus in mice. PLoS One 6:e28901. doi:10.1371/journal.pone.0028901 22194944 PMC3241702

[29] Roces CB, Lou G, Jain N, Abraham S, Thomas A, Halbert GW, Perrie Y. 2020. Manufacturing considerations for the development of lipid nanoparticles using microfluidics. Pharmaceutics 12:1095. doi:10.3390/pharmaceutics12111095 33203082 PMC7697682

[30] Halbur PG, Paul PS, Frey ML, Landgraf J, Eernisse K, Meng XJ, Lum MA, Andrews JJ, Rathje JA. 1995. Comparison of the pathogenicity of two US porcine reproductive and respiratory syndrome virus isolates with that of the Lelystad virus. Vet Pathol 32:648–660. doi:10.1177/030098589503200606 8592800

[31] Gauger PC, Loving CL, Khurana S, Lorusso A, Perez DR, Kehrli ME, Roth JA, Golding H, Vincent AL. 2014. Live attenuated influenza A virus vaccine protects against A(H1N1)pdm09 heterologous challenge without vaccine associated enhanced respiratory disease. Virology 471–473:93–104. doi:10.1016/j.virol.2014.10.003 25461535

[32] Russo RC, Garcia CC, Barcelos LS, Rachid MA, Guabiraba R, Roffê E, Souza ALS, Sousa LP, Mirolo M, Doni A, Cassali GD, Pinho V, Locati M, Teixeira MM. 2011. Phosphoinositide 3-kinase γ plays a critical role in bleomycin-induced pulmonary inflammation and fibrosis in mice. J Leukoc Biol 89:269–282. doi:10.1189/jlb.0610346 21048214

[33] Tang Y, Wang Z, Huo C, Guo X, Yang G, Wang M, Tian H, Hu Y, Dong H. 2018. Antiviral effects of Shuanghuanglian injection powder against influenza A virus H5N1 in vitro and in vivo. Microb Pathog 121:318–324. doi:10.1016/j.micpath.2018.06.004 29864534

[34] Zhang W, Pfeifle A, Lansdell C, Frahm G, Cecillon J, Tamming L, Gravel C, Gao J, Thulasi Raman SN, Wang L, Sauve S, Rosu-Myles M, Li X, Johnston MJW. 2023. The expression kinetics and immunogenicity of lipid nanoparticles delivering plasmid DNA and mRNA in mice. Vaccines (Basel) 11:1580. doi:10.3390/vaccines11101580 37896985 PMC10610642

[35] Pfeifle A, Thulasi Raman SN, Lansdell C, Zhang W, Tamming L, Cecillon J, Laryea E, Patel D, Wu J, Gravel C, Frahm G, Gao J, Chen W, Chaconas G, Sauve S, Rosu-Myles M, Wang L, Johnston MJW, Li X. 2023. DNA lipid nanoparticle vaccine targeting outer surface protein C affords protection against homologous Borrelia burgdorferi needle challenge in mice. Front Immunol 14:1020134. doi:10.3389/fimmu.2023.1020134 37006299 PMC10060826

[36] Mucker EM, Karmali PP, Vega J, Kwilas SA, Wu H, Joselyn M, Ballantyne J, Sampey D, Mukthavaram R, Sullivan E, Chivukula P, Hooper JW. 2020. Lipid nanoparticle formulation increases efficiency of DNA-vectored vaccines/immunoprophylaxis in animals including transchromosomic bovines. Sci Rep 10:8764. doi:10.1038/s41598-020-65059-0 32472093 PMC7260227

[37] Liao HC, Shen KY, Yang CH, Chiu FF, Chiang CY, Chai KM, Huang WC, Ho HM, Chen YH, Huang MS, Liao CL, Chen HW, Huang MH, Liu SJ. 2024. Lipid nanoparticle-encapsulated DNA vaccine robustly induce superior immune responses to the mRNA vaccine in Syrian hamsters. Mol Ther Methods Clin Dev 32:101169. doi:10.1016/j.omtm.2023.101169 38187094 PMC10767207

[38] Guimaraes LC, Costa PAC, Scalzo Júnior SRA, Ferreira HAS, Braga ACS, de Oliveira LC, Figueiredo MM, Shepherd S, Hamilton A, Queiroz-Junior CM, da Silva WN, da Silva NJA, Rodrigues Alves MT, Santos AK, de Faria KKS, Marim FM, Fukumasu H, Birbrair A, Teixeira-Carvalho A, de Aguiar RS, Mitchell MJ, Teixeira MM, Vasconcelos Costa V, Frezard F, Guimaraes PPG. 2024. Nanoparticle-based DNA vaccine protects against SARS-CoV-2 variants in female preclinical models. Nat Commun 15:590. doi:10.1038/s41467-024-44830-1 38238326 PMC10796936

[39] Kiseleva I, Larionova N, Kuznetsov V, Rudenko L. 2010. Phenotypic characteristics of novel swine-origin influenza A/California/07/2009 (H1N1) virus. Influenza Other Respir Viruses 4:1–5. doi:10.1111/j.1750-2659.2009.00118.x PMC4941948 20021501

[40] Kodihalli S, Goto H, Kobasa DL, Krauss S, Kawaoka Y, Webster RG. 1999. DNA vaccine encoding hemagglutinin provides protective immunity against H5N1 influenza virus infection in mice. J Virol 73:2094–2098. doi:10.1128/JVI.73.3.2094-2098.1999 9971791 PMC104453

